# Implementation of Accurate Parameter Identification for Proton Exchange Membrane Fuel Cells and Photovoltaic Cells Based on Improved Honey Badger Algorithm

**DOI:** 10.3390/mi15080998

**Published:** 2024-07-31

**Authors:** Wei-Lun Yu, Chen-Kai Wen, En-Jui Liu, Jen-Yuan Chang

**Affiliations:** 1 Department of Power Mechanical Engineering, National Tsing Hua University, Hsinchu 30013, Taiwan; s105033810@m105.nthu.edu.tw; 2 Mechanical and Mechatronics Systems Research Laboratories, Industrial Technology Research Institute, Hsinchu 310401, Taiwan; 3 Department of Green Energy and Information Technology, National Taitung University, Taitung 95092, Taiwan; 11022113@gm.nttu.edu.tw

**Keywords:** PEM fuel cell, photovoltaic cell, improved honey badger algorithm, metaheuristic algorithm, parameter identification

## Abstract

Predicting the system efficiency of green energy and developing forward-looking power technologies are key points to accelerating the global energy transition. This research focuses on optimizing the parameters of proton exchange membrane fuel cells (PEMFCs) and photovoltaic (PV) cells using the honey badger algorithm (HBA), a swarm intelligence algorithm, to accurately present the performance characteristics and efficiency of the systems. Although the HBA has a fast search speed, it was found that the algorithm’s search stability is relatively low. Therefore, this study also enhances the HBA’s global search capability through the rapid iterative characteristics of spiral search. This method will effectively expand the algorithm’s functional search range in a multidimensional and complex solution space. Additionally, the introduction of a sigmoid function will smoothen the algorithm’s exploration and exploitation mechanisms. To test the robustness of the proposed methodology, an extensive test was conducted using the CEC’17 benchmark functions set and real-life applications of PEMFC and PV cells. The results of the aforementioned test proved that with regard to the optimization of PEMFC and PV cell parameters, the improved HBA is significantly advantageous to the original in terms of both solving capability and speed. The results of this research study not only make definite progress in the field of bio-inspired computing but, more importantly, provide a rapid and accurate method for predicting the maximum power point for fuel cells and photovoltaic cells, offering a more efficient and intelligent solution for green energy.

## 1. Introduction

There are many existing studies that clearly demonstrated the adverse outcomes of the greenhouse effect, namely global warming, rising sea levels and an increased frequency in the occurrence of extreme weather events.

The life-altering transformation is now a pressing global issue that must be addressed, with countries putting forward various relevant courses of action for net-zero emissions, with the hope of achieving environmental sustainability by 2050. One of the key strategies proposed hinges on renewable energy systems—according to the Electricity Market Report 2023 by the International Energy Agency (IEA), renewable energy will overtake coal as the world’s largest source of electricity. Although renewable energy can significantly reduce greenhouse gas emissions, it has the issue of intermittent energy supply. This results in a spatial and temporal gap between the user side and the power supply side [1]. To stabilize the grid frequency, it is essential to develop green energy systems that can provide a stable power supply, such as proton exchange membrane fuel cells (PEMFCs).

PEMFCs are a type of fuel cell that uses hydrogen fuel and air to generate electricity and heat. With zero emissions, efficient energy conversion, a fast start-up time and a wide range of operating temperatures, these cells see widespread usage in many fields, namely portable power, backup power, household stationary power generation and the automobile industry. The PEMFC model is a complex system that is characterized by its nonlinearity and multivariable, strong coupling—when operating, factors such as the environmental and system temperature, humidity, energy density and fuel inlet pressure will affect its output efficacy. Consequently, module improvement and system control are critical in improving the current fuel cell technology.

The output of PEMFCs can be represented by a nonlinear curve. Currently, there are three types of fuel cell models used for effective development and analysis of the cell’s system characteristics for consequent evaluations and applications. Namely, these models are mechanistic models [2,3], analytical models [4] and semi-empirical models [5]. The mechanistic model is also known as a theoretical model. It makes use of differential and algebraic equations to represent the physical and electrochemical processes in the system and often uses electrochemical impedance spectroscopy to conduct spectral analysis. When converted into equivalent circuit models [6], it can be further subcategorized into single-domain models and multi-domain models.

The analytical model is suitable for fast calculations—it derives relevant equations through the relationship between voltage and current density. However, one disadvantage of analytical models is that they cannot provide an accurate representation of the internal workings of the system. One such model is the black-box model [7], which is a system that derives input–output relationships through statistical data—with artificial neural networks [8], adaptive neuro-fuzzy inference systems [9] and support vector machines [10] being some of the frequently used methods. Lastly, the semi-empirical model is a hybrid model that combines the derived electrochemical equations with empirical equations. However, it must be noted that even though the model can provide effective performance simulations and accurate performance predictions, there are many undetermined parameters in it that must be obtained experimentally.

The accuracy of PEMFC modeling will significantly affect its performance evaluation [11], optimal control [12], the cell’s maximum power point tracking control [13] and the degradation adaptive energy management strategy [14]. As such, the parameter estimation of the PEMFC is a vital factor. Presently, there are a series of parametric analysis techniques used in achieving modeling accuracy, such as the parametric analysis method [15], nonlinear least-square method [16], current switching method and electrochemical impedance spectroscopy [17]. Following the rapid advancement of computing power and artificial intelligence, the application of swarm intelligence algorithms to fuel cells has also demonstrated good results. Swarm intelligence algorithms are based on swarms of living organisms, using the nature of division of labor and cooperation found in these groups to seek optimal solutions in the given search space. The algorithm will search for the parameter values to be determined and later insert the identified parameter values into the optimized model to achieve accurate modeling. Some examples of swarm intelligence algorithms include the genetic algorithm [18], particle swarm optimization [19], artificial bee colony [20], whale optimization algorithm [21] and grey wolf optimizer [22].

With regard to the control of PV systems, manufacturers generally do not provide detailed parameters of the PV cell, thus making the testing and prediction of the system performance a problem. Therefore, many studies have been conducted in order to identify the parameters of PV cells. Following this, the current mainstream battery modules and the relevant methods and technologies for obtaining system parameters will be explained accordingly.

Firstly, numerical methods using numerical analysis are commonly used to identify system parameters of diode modules. Some of these methods include the linear least-squares method [23], the Levenberg–Marquardt algorithm [24] and the curve fitting method. For these methods, the accuracy of the parameters identified will increase with the increase in the number of known data points provided as references. Currently, the percentage error for the accuracy of parameter estimation falls between the range of 90.5 and 99%. However, there are certain disadvantages of numerical methods. For one, they are particularly sensitive to initial conditions, which may result in solutions that are local optima. They also require a long computation time due to the large amount of data needed for calibration.

The second method is a mathematical model based on establishing mathematical equations from the three main characteristics of the battery—the open-circuit voltage (OCV), short-circuit current and maximum power point (MPP) [25]. Additionally, parameter identification is performed under standardized test conditions and variable weather conditions. This modeling approach is quick and simple, seeing as how it only requires the manufacturer to provide measurements of the three aforementioned characteristics to be able to create the model. Thereafter, by solving differential equations, the parameters of the system module can be obtained. The disadvantage of this method, however, is that the nonlinearity of the PV module, alongside the issue of transcendental functions, is disregarded in the model.

The third method is the use of metaheuristic algorithms, which have been proven in many previous studies to effectively ameliorate the disadvantages of the two previously discussed approaches, such as the sensitivity to initial conditions and long computational time. The convergence speed, reliability and accuracy of this method have significant improvements over the previous two methods due to the search mechanism of the algorithm being more comprehensive. By establishing logical mathematical models, many engineering problems can also be solved. However, this method comes with its own drawbacks as well. The search mechanism of the various metaheuristic algorithms can lead to a wide range of computational errors, with a mean absolute percentage error between the range of 78% and 98.6%. Presently, there are many published results on the application of swarm intelligence algorithms in green energy systems. Some of the algorithms that were used are as follows: artificial neural networks [26], particle swarm optimization, artificial bee colony [27], cuckoo search [28], the whale optimization algorithm [29], the firefly algorithm [30] and the flower pollination algorithm [31].

Concerning the solving of optimization problems, many scholars have attempted to use the logical reasoning of AI machine learning to propose different heuristics methods as an alternative to traditional algorithms, which take a long time to find solutions. In practical applications of AI, biomimetic evolution and social behavior are often simulated to carry out algorithmic innovation. These biomimetic evolutionary computations are also known as metaheuristic algorithms. Glover coined the term “meta-heuristic” in 1986 [32], referring to a generalized heuristic algorithm that is able to solve different types of optimization problems. There are two major search mechanisms in a metaheuristic algorithm: exploitation and exploration. The former focuses on searching for local optima, while the latter explores new search regions for global optima. Metaheuristic algorithms are highly adaptive and can obtain the closest approximate solution in an efficient and timely manner. This attribute makes them suitable for addressing nonlinear and high-dimensional complex problems. Nevertheless, there is no algorithm that will be able to deal with every optimization problem, leading to extensive academic research on the robustness testing of algorithms in recent years. In the multidimensional search space, the process by which an algorithm identifies potential regions can be divided into exploration and exploitation. Exploration refers to the algorithm searching for solutions in previously unexplored areas, generating new optimal solutions and increasing diversity. Exploitation, on the other hand, involves the algorithm focusing on known promising regions to find the best solutions, thus accelerating convergence.

The difference between exploration and exploitation lies in their objectives: exploration aims at discovering new areas and solutions, while exploitation concentrates on optimizing the currently identified best solutions. The quality of the solution depends on the balance between these two search behaviors. Excessive exploration can hinder the progress of exploitation, whereas excessive exploitation can reduce diversity, potentially leading to local optima. Therefore, finding the right balance between exploration and exploitation in metaheuristic algorithms is a significant challenge. Robustness refers to the ability of an algorithm to continue operating normally and obtain satisfactory solutions despite facing errors or deviations. The majority of the optimization problems used for robustness testing feature a continuous or discrete multi-objective variable design. Because energy systems are nonlinear and complex, there will be difficulty in terms of system control and prediction. A highly robust metaheuristic algorithm is therefore needed to prevent large errors in the characteristic curve.

The next section will describe in detail the approach towards the modeling of green energy systems, with the addition of the improved honey badger algorithm. The overview and contributions of the study are as follows:

- Establishing the model for green energy systems (for PEMFC and PV cells).
- By adjusting the search weight ratio of the HBA algorithm using the sigmoid function and modifying the honey phase search mechanism of HBA into a spiral form, the global search efficiency of HBA is significantly enhanced.
- Conducting robustness testing of the algorithm using the CEC’17 benchmark functions set.
- Using the improved honey badger algorithm to implement highly accurate parameter identification in green energy systems.

This research focuses primarily on the optimization of green energy system model parameters. Numerous studies [33,34,35] indicate that in future smart cities with microgrids, photovoltaic cells and hydrogen fuel cells will be the primary sources of green energy. If the system characteristics can be accurately represented, it will be possible to predict system performance under different testing conditions. For manufacturers, maximum power point control can be more precise, and system aging indicators can be established from model parameters.

## 2. Methodology

### 2.1. Proton Exchange Membrane Modeling and Theory

**Figure 1:**
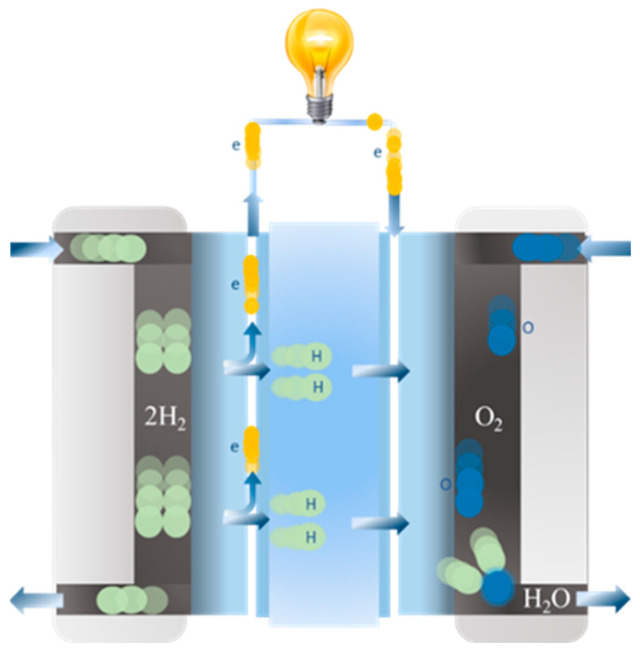
PEM fuel cell system diagram [36].

The catalyst layers, the membrane and the gas diffusion layers make up the important components of the PEMFC. Hydrogen fuel is processed at the anode, while oxygen is provided at the cathode. As hydrogen enters the flow channel, it passes through the gas diffusion layer and is distributed into the catalyst layer. The catalyst will then separate the hydrogen gas into hydrogen ions and electrons. The hydrogen ions and electrons are transferred to the cathode’s reaction site, with the ions passing through the proton exchange membrane and the electrons passing through the current collector via the external circuit. Finally, oxygen at the cathode diffuses into the catalyst later and reacts with the hydrogen ions and electrons to produce water. Figure 1 shows the operation diagram of the fuel cell, while Equations (1)–(3) show the oxidation reaction, reduction reaction and redox reaction of the fuel cell, respectively.

(1) $$H_{2}\to 2e^{-}+2H^{+}$$

(2) $${2e}^{-}+2H^{+}+\frac{1}{2}O_{2}\to H_{2}O$$

(3) $$H_{2}+\frac{1}{2}O_{2}\to H_{2}O$$

Fuel cell efficiency is a nonlinear output curve. During system operation, the mass and charge will encounter transfer resistance, thus resulting in the cell voltage not being able to reach its theoretical value. The model uses the Nernst Equation for calculating the standard cell potential in the system. The partial pressure of the reactants in the inlet flow channel varies according to the partial pressures of hydrogen and oxygen, and the partial pressure of water vapor in the channel is defined by the saturated vapor pressure at the operating temperature of the fuel cell, as expressed in Equation (4). $P_{O_{2}}$ and $P_{H_{2}}$ represent the inlet pressures of oxygen and hydrogen.

(4) $$E_{Nernst}=1.229-0.85\times 10^{-3}\left(T-298.15\right)+4.3085\times 10^{-5}\times T\left[ln\left(P_{H_{2}}\right)+0.5ln\left(P_{O_{2}}\right)\right]$$

According to the Maxwell–Stefan equation, the partial pressures of hydrogen and oxygen, and the saturated vapor pressure can be expressed as follows. The function of temperature as a function of saturated vapor pressure of water and oxygen is expressed in Equations (5) and (6). In the equation, $P_{H_{2}O}$ is the pressure of saturated water, ${RH}_{C}$ and ${RH}_{a}$ are the relative humidity of the cathode and anode, and $P_{C}$ and $P_{a}$ are the inlet pressures of the cathode and anode, respectively.

(5) PO2=RHC×PH2O1exp4.192×iAT1.334RHCPH2OPC−1

(6) PH2=0.5RHa×PH2O1exp1.635×iAT1.334RHaPH2OPa−1

**Figure 2:**
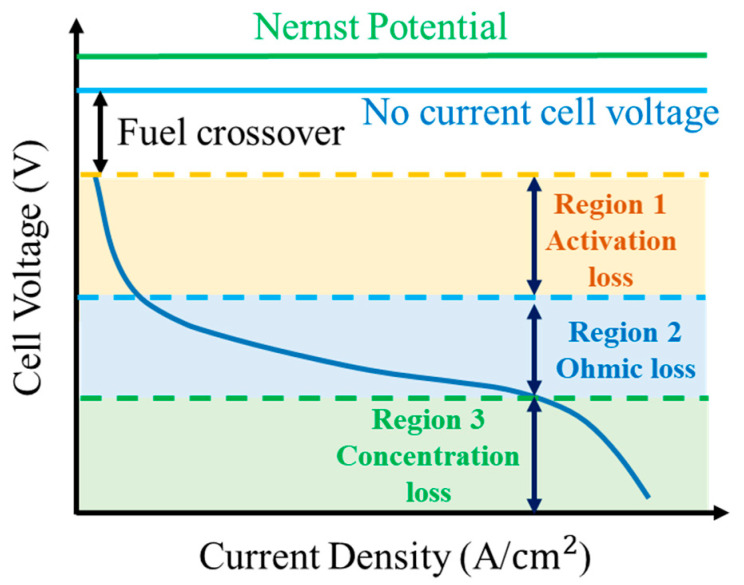
PEM fuel cell polarization curve diagram [36].

As shown in Figure 2, the voltage loss during the discharge process of the fuel cell is largely affected by activation polarization, ohmic polarization and concentration polarization. Activation polarization is a result of delayed electrochemical reactions on the electrode surface, leading the cell potential to deviate from the equilibrium potential. This polarization tends to occur at low current density. In this section, the Butler–Volmer equation is used to describe the electrostatic potential of the reaction.

The Tafel equation describes the relationship between the overpotential of the half-reaction and current density. The Tafel equation is used to calculate the overpotentials of the anode and cathode, before adding them together to find out the activation overpotential of the whole cell, as expressed in Equation (7).

(7) $$\eta_{act}=\eta_{anode}+\eta_{cathode}=\zeta_{1}+\zeta_{2}T+\zeta_{3}T\left[ln\left(c_{O_{2}}\right)\right]+\zeta_{4}T\left[ln\left(i\right)\right]$$

*ζ*_1_, *ζ*_2_, *ζ*_3_, and *ζ*_4_ represent the four parameters in the activation overpotential equation. Subsequent to the above equation, Henry’s law is used to solve for *c*_*o*_2__, as shown in Equation (8). By writing the molar volume of oxygen concentration as a relationship between the temperature and partial pressure of gas, and then inserting the results of the calculation into Equation (7), the overpotential generated by activation polarization can be determined.

(8) $$c_{o_{2}}=\frac{P_{O_{2}}}{5.08\times 10^{6}}\cdot exp\left(\frac{498}{T}\right)$$

Ohmic polarization predominantly describes the resistance generated during the movement of ions and electrons. There are two causes for the aforementioned resistance—resistance generated when hydrogen ions pass through the proton exchange membrane and resistance generated by the transfer of electrons through the current collector or at the electrodes, as expressed in Equations (9) and (10). Of the two, the resistance generated by the hydrogen ions is the most important factor in ohmic polarization. Equation (11) is expressed as the expansion of *R_M_* during ionic transfer, calculated with the law of resistance, where $\rho_{M}$ is the resistivity of the proton exchange membrane, *L* is the length of the membrane, *A* is the area of the membrane, *λ* is the correction parameter and DuPont’s Nafion membrane is used for the calculation of resistivity. Equation (12) shows the numerical expression for the resistivity of the Nafion membrane.

(9) $$V_{ohmic}=V_{ohmic,\;electronic}+V_{\mathrm{ohmic},\;\mathrm{proton}}$$

(10) $$V_{ohmic}=i\left(R_{C}+R_{M}\right)$$

(11) $$R_{M}=\rho_{M}\frac{L}{A}$$

(12) ρM=181.6×1+0.03×iA+0.062×T303iA2.5λ−0.634−3×iAexp4.18×T−303T

Concentration polarization describes the loss of potential due to the mass transport resistance of reactants and occurs mainly at high current densities. When fuel cells generate electricity, reactants near the electrode will be constantly consumed. Once the transfer rate of the reactant becomes smaller than its consumption rate, there will be a drop in the concentration at the reaction site, leading to potential loss. This polarization is expressed in Equation (13), where *i_max_* is the maximum current density of the system and b is the parameter after simplification.

(13) $$V_{con}=-b\times ln\left(1-\frac{i}{i_{max}}\right)$$

In most cases, sites with low current density are affected mainly by activation polarization. As the current density increases to a moderate level, ohmic polarization becomes the main cause for the cell potential loss. At a high current density, the main cause of potential loss becomes concentration polarization. When the cell is under open-circuit conditions, where no net current is generated, the voltage of the fuel cell is defined as open-circuit voltage. When the output current of the system flows externally, polarization occurs—the relationship between that and the fuel cell system is expressed in Equation (14), where *n_cell_* is the number of single cells in the system and *V_stack_* is the voltage value of the fuel cell stack.

(14) $$V_{Stack}=n_{Cell}\times \left(E_{Nernst}-V_{act}-V_{ohmic}-V_{con}\right)$$

### 2.2. Photovoltaics Cells Modeling and Theory

**Figure 3:**
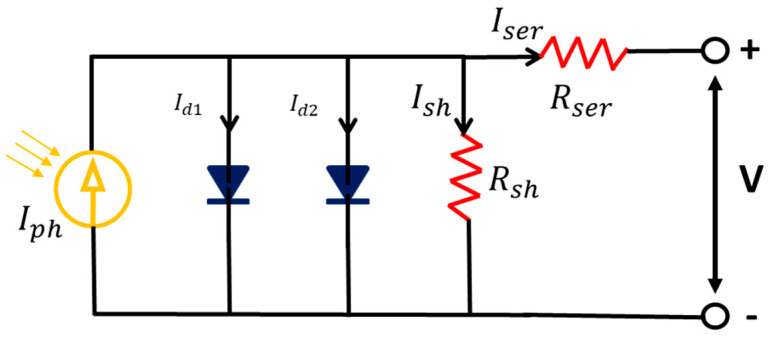
Photovoltaic circuit diagram [27].

Photovoltaic cells, also known as solar cells, operate on the principles of the photovoltaic effect—voltage and current are generated in the battery components via light or electromagnetic radiation. Silicon solar cells in commercial usage are based on carrier diffusion and recombination at the internal P-N junction. As the P-N junction reaches equilibrium, a depletion region is formed around the interface. The electrons and holes in the depletion region will be conducted to the electrode via diffusion, thus generating energy. Still, the output strength of PV cells can be lost due to system or contact resistances causing current loss. Equation (15) shows the circuit model of a PV cell, where *I_ph_* is the photogenerated current, *I*_*d*1_ and *I*_*d*2_ are the diffusion and recombination current of the diode, respectively, and *I_sh_* is the leakage current caused by the shunt group *R_sh_*. So as to accurately establish the model of the PV cell, this study adopts the Shockey diode equation to calculate the relationship between the currents *I*_*d*1_ and *I*_*d*2_ with the reverse saturation current, expressed, respectively, in Equations (16) and (17). The double-diode circuit of the PV is shown in Figure 3.

(15) $$I_{t}=I_{ph}-I_{d1}-I_{d2}-I_{sh}$$

(16) $$I_{d1}=I_{sd1}\left[exp\left(\frac{q\left(V_{t}+R_{ser}\times I_{t}\right)}{n_{1}\times k\times T}\right)-1\right]$$

(17) $$I_{d2}=I_{sd2}\left[exp\left(\frac{q\left(V_{t}+R_{ser}\times I_{t}\right)}{n_{2}\times k\times T}\right)-1\right]$$

(18) $$I_{sh}=\frac{V_{t}+R_{ser}\times I_{t}}{R_{sh}}$$

Therein, *Vt* is the terminal voltage, *q* represents the charge of a single electron (as measured in coulombs), *k* is the Boltzmann constant, and T is the battery temperature. In total, the double diode (DD) model has seven undetermined parameters: (i) *I_ph_*—the current generated by light, (ii) *I*_*sd*1_—the reverse saturation current of diffusion, (iii) *I*_*sd*2_—the reverse saturation current of recombination, (iv) *n*_1_—the ideality factor of the diffusion diode, (v) *n*_2_—the ideality factor of the recombination diode, (vi) *R_ser_*—the series resistance and (vii) *R_sh_*—the shunt resistance. The estimation methods for the abovementioned parameters can be derived from the PV cells Equations (15)–(18).

### 2.3. Honey Badger Algorithm and Its Improvements

The Honey Badger Algorithm (HBA) is a swarm intelligence optimization algorithm proposed by Hashim et al. in 2022 [37]. The mathematical model of the algorithm is inspired by the foraging behaviors of honey badgers—simulating their static and dynamic search behaviors. In the following, the search mechanism of the HBA will be explained.

(19) $$A=\left[\begin{array}{c} x_{1}\\ x_{2}\\ \ldots \\ x_{n} \end{array}\right]=\left[\begin{array}{cc} \begin{array}{cc} x_{11} & x_{12}\\ x_{21} & x_{22} \end{array} & \begin{array}{ccc} x_{13} & \ldots & x_{1D}\\ x_{23} & \ldots & x_{2D} \end{array}\\ \begin{array}{cc} & \ldots \\ x_{n1} & x_{n2} \end{array} & \begin{array}{ccc} \ldots & \ldots & \\ x_{n3} & \ldots & x_{nD} \end{array} \end{array}\right]$$

*A* represents the entire population of the honey badgers, *x_i_* represents the location of the *i_th_* honey badger, *n* represents the number of honey badgers, *D* represents the dimension, and *r*_1_ is a number between [0,1] that is randomly generated within the upper bounds (UB) and lower bounds (LB) to represent the coordinates of the *i_th_* honey badger, *S* refers to the squared distance between honey badgers and *d_i_* refers to the distance between the best solution and the honey badger.

(20) $$x_{i}={lb}_{i}+r_{1}*\left({ub}_{i}-{lb}_{i}\right)$$

(21) $$I_{i}=r_{2}*\frac{S}{4\pi d_{i}^{2}}$$

The algorithm determines the distance between the honey badger and its prey (represented as *x_prey_*) through smell intensity (represented as *I_i_*). As the honey badger gets closer to the prey, the distance (*d_i_*) will decrease, and the smell intensity will increase. *S* denotes the source intensity, also known as concentration intensity, which indicates the density of the honey badgers. *Di* denotes the distance between the prey and the *i_th_* badger. There are two key factors influencing *I_i_*—first, the distance between the honey badger and prey and, secondly, the distance between honey badgers. Note that *I_i_* is inversely proportional to *d_i_* and proportional to *S*.

(22) $$\alpha ={Ce}^{\left(\frac{-t}{t_{max}}\right)},\;C=2$$

The density factor *α* is the transition factor that governs the algorithm processes of exploration to exploitation, where *t_max_* represents the maximum number of iterations and *C* is a constant value of 2. Reducing the randomness of the density factor over time will allow the algorithm to have an improved global search capability at the start of the iterations. It also reduces random disturbances and improves local search capability in later iterations.

**Figure 4:**
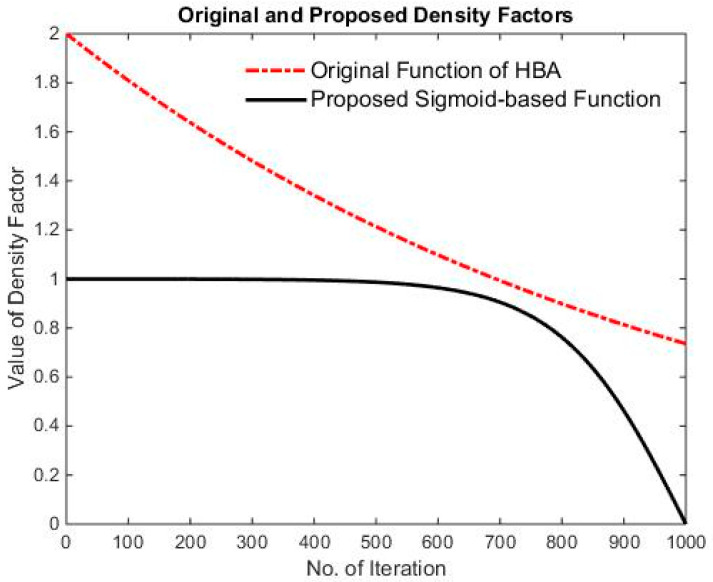
Proposed improvements and iteration of the original density factor.

In the original algorithm, the density factor incorporates both global and local search mechanisms within two search formulas. However, the digging phase accounts for a heavier proportion in the local search mechanism. Consequently, to smoothen the algorithm transition, this study plans to use the sigmoid function as a conversion factor. The sigmoid function has good gradient properties that allow for a faster computation speed, as shown in Equation (23). The conversions of the two transition factors can be seen in Figure 4.

(23) $$S(t)=\frac{1}{1+e^{-\left(1-\frac{t}{t_{max}}\right)}}$$

**Figure 5:**
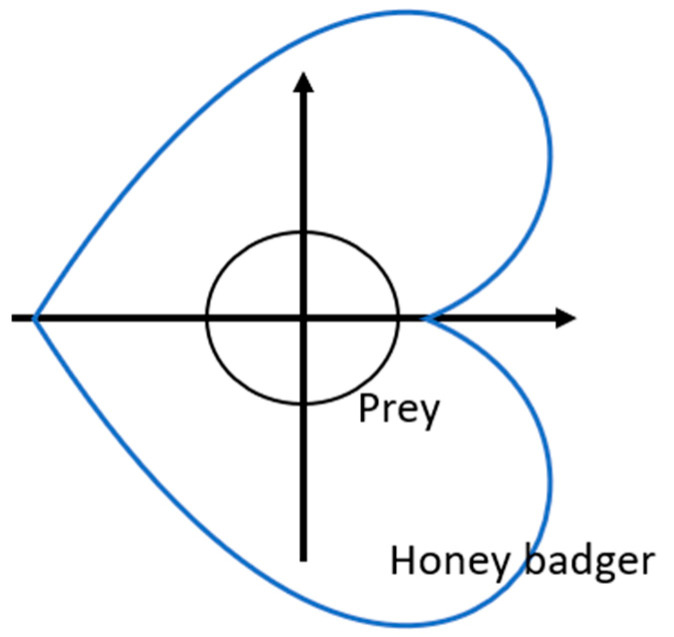
Diagram of heart-shaped search during the digging phase.

Digging phase:

In the digging phase, the honey badger performs a search whose trajectory follows the shape of a cardioid, as shown in Figure 5, which simulates the search trajectory of the honey badger along a specific radius produced by the prey. This trajectory is expressed in Equation (24), where *x_prey_* represents the prey location, *β* represents the honey badger’s ability to hunt for food, *d_i_* represents the distance between the *i_th_* honey badger and the prey, and *r*_3_, *r*_4_, and *r*_5_ are random numbers between [0,1]. *F* represents the direction of the search, which is used by the HBA to avoid obtaining a local optimal solution.

(24) $$x_{new}=x_{prey}+F\times \beta \times I\times x_{prey}+F\times r_{3}\times \alpha \times d_{i}\times \left|\cos\left(2\pi r_{4}\right)\times \left[1-\cos\left(2\pi r_{5}\right)\right]\right|$$

(25) $$F=\left\{\begin{array}{c} 1,\;if\;r_{6}\leq 0.5\\ -1,\;else \end{array}\right.$$

Honey phase:

In the honey phase, the honey badger follows honeyguide birds to be guided directly to the location of the prey.

(26) $$x_{new}=x_{prey}+F\times r_{7}\times \alpha \times d_{i}$$

*r*_7_ is a random number in the range of [0,1], *x_new_* represents the new location of the honey badger, *x_prey_* represents the prey location, and *F* is the same as in Equation (25): a parameter used to modify the search direction. In this phase, a medium-short distance advancement in any direction from the existing optimal solution is executed while simultaneously performing local and global searches.

(27) $$x_{new}=x_{prey}+F\times \alpha \times d_{i}\times e^{bl_{1}}\times cos\left(2\pi l_{2}\right)$$

So as to enhance the global search capability of the algorithm, the medium-distance search in the honey phase search mechanism is upgraded to a spiral search. This is expressed in Equation (27), where *l*_1_ and *l*_2_ are random numbers in the range of [−1,1].

### 2.4. Parameter Settings of Algorithms in Various Optimization Problems

When evaluating the robustness of metaheuristic algorithms, the CEC benchmark is considered the premier platform for comparing stochastic search algorithms. The CEC competition functions are widely used for benchmarking the performance of advanced algorithms. The CEC’17 test suite comprises 29 mathematical functions, which are divided into unimodal functions (F1–F3, except F2), multimodal functions (F4–F10), hybrid functions (F11–F20), and composition functions (F21–F30) [38]. These highly complex functions are essential for assessing the stability and effectiveness of algorithms in solving optimization problems. This study uses the CEC’17 benchmark functions [39] set to conduct robustness testing of the improved HBA. Presently, this is the most commonly used test set—it has 30 sets of complex mathematical models that can effectively evaluate the algorithm performance. The parameters of the HBA are set as follows: the number of independent operations (Runtime#) is 30, the number of iterations (Iteration#) is 1000, and the number of honey badgers (Agent#) is 30, as shown in Table 1.

In the following, the parameter settings for the hydrogen fuel cell model are expressed in Equation (28), with *F*(*X*) denoting the objective function. The identification of the physical parameters *X* = [*ξ*_1_ *ξ*_2_ *ξ*_3_ *ξ*_4_ *λ b*] will significantly influence the results of the model—these unknown values need to be accurately determined in order to calculate the practical voltage–current (V-I) characteristic curve of the cell. In this study, the objective function *F*(*X*) is used to find an optimal set of parameter values so as to minimize the sum of squares error (SSE) of both the experimental voltage *V*_*exp*,*fc*_ and the estimated model voltage as calculated from the equation *V*_*mod*,*fc*_. Table 2 lists the scope of optimization searches of the parameters to be determined in the NedStackPS6 PEMFC.

(28) $$F\left(X\right)=min\sum {\left[V_{exp,\;fc}\left(X\right)-V_{mod,\;fc}\left(X\right)\right]}^{2}$$

For the PV cell, the objection function of the DD model is expressed in Equation (28), with seven undetermined parameters in the PV model that will be solved by two types of HBAs in this study. These parameters are as follows: series resistance (*R_ser_*), shunt resistance (*R_sh_*), the photocurrent of the cell (*I_ph_*), reverse saturation currents of the diode (*I*_*sd*1_ and *I*_*sd*2_) and the ideality factors of the diode (*n*_1_ and *n*_2_). The root-mean-square error is used as the objective function G(X), as expressed in Equation (29), where *I*_*exp*,*pv*_ is the experimental value of the cell, *I*_*mod*,*pv*_ is the current value after calculation, and *n* is the number of experimental data samples. Table 3 shows the PV cell parameter setting boundary.

(29) $$G\left(X\right)=min\sqrt{\frac{\sum {\left(I_{exp,\;pv}-I_{mod,\;pv}\right)}^{2}}{n}}$$

## 3. Results and Discussion

In this study, the CEC’17 benchmark functions test set is used to conduct a score-based robustness comparison, where 1 point will be awarded to the winner in each test. For any ties, both are attributed 1 point. The best fitness, mean fitness and standard deviation are each evaluated separately before combining the total score of the three. The function test performances are shown in Table 4. As seen, the original HBA performs better with regard to standard deviation, scoring 17 points. However, the improved HBA surpasses the original when comparing best fitness and mean fitness, scoring 18 points and 17 points, respectively.

**Figure 6:**
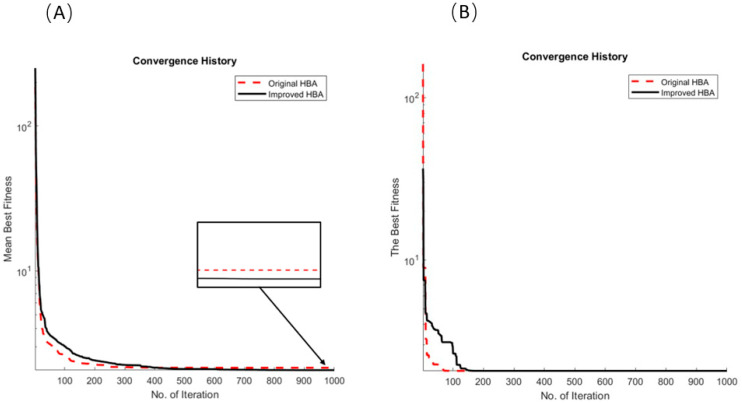
IHBA and HBA for PEMFC identification of (**A**) mean best fitness and (**B**) the best fitness.

**Figure 7:**
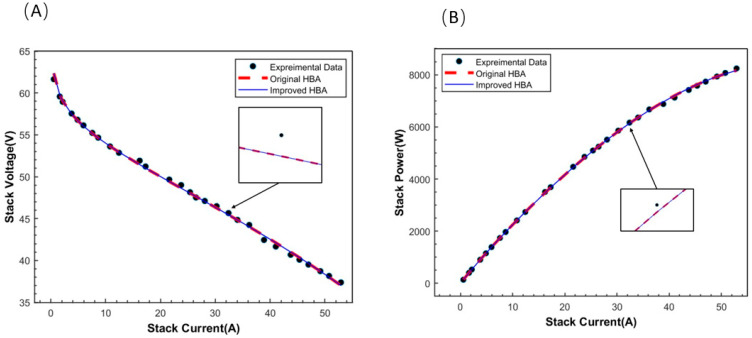
IHBA and HBA for PEMFC identification of (**A**) I-V curves and (**B**) I-P curves.

Through the robustness test, it is proven that the improved algorithm shows enhancement in both its search capability and search speed. Subsequently, the results of the application of the two versions of HBA to PEMFCs will be discussed and compared to seven other algorithms. Table 5 shows the fuel cell parameters and the best fitness of the various algorithms. Table 6 shows the comparison between the calculated and experimental terminal voltage values of the two HBAs. Figure 6A,B show the results of the best fitness and mean fitness results of the two HBAs, with the iterative speed clearly demonstrating the ability of the sigmoid function to accelerate the search speed of the algorithm. Figure 7A shows the current versus the voltage of the fuel cell stack, while Figure 7B shows the current versus the power of the fuel cell stack—the black dots represent the experimental values, the red line represents the original HBA, and the blue line represents the improved HBA. Not only is the computational efficiency of the improved HBA better than the original HBA but it also beats out many other algorithms.

**Figure 8:**
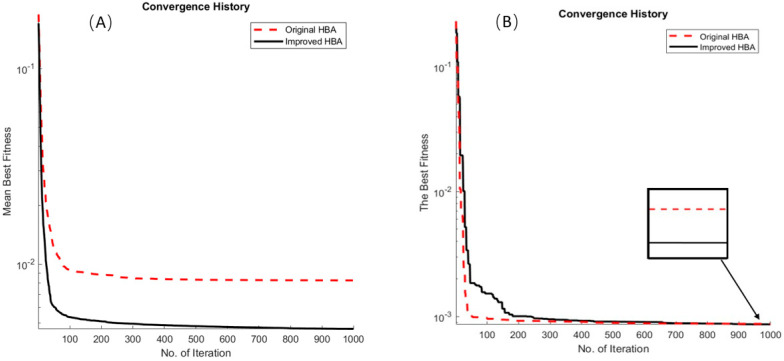
Identification of photovoltaic cell parameters by IHBA and HBA: (**A**) mean best fitness convergence history and (**B**) the best fitness convergence history.

**Figure 9:**
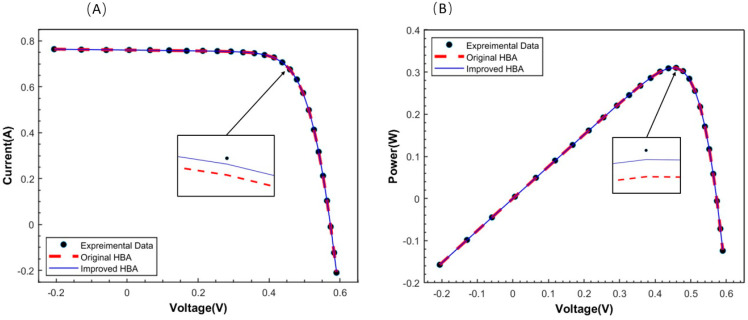
Experimental data for photovoltaic cell, and calculations by IHBA and HBA of (**A**) V-I curve and (**B**) V-P curve.

For the results of parameter identification in PV cells, Table 7 shows the seven parameters of the PV cell and the best fitness, Table 7 shows the best parameters and the best fitness of the various algorithms, and Table 8 shows the comparison of the calculated and experimental terminal current values of the two HBA algorithm. Figure 8A,B show the iterative graph of the two HBAs, with two types of convergent solutions: mean best solution and best solution. The results of the improved HBA in terms of its mean fitness demonstrate a significant improvement over the original algorithm. Figure 9A shows the I–V characteristic curve analysis results of the original HBA and the improved HBA as applied to the DD model. From the analysis results, it is clear that the calculated values of the improved HBA are relatively close to the experimental values. Figure 9B shows the power-V characteristic curve analysis results of the two HBAs.

## 4. Conclusions

This study proposed an improved honey badger algorithm by modifying the original’s algorithm search criteria during the position update of the honey phase. A spiral search is added to improve the algorithm’s accuracy in finding the optimal solution, and a sigmoid function is used to smoothen the algorithm transition from global to local search. Both the original and improved HBA are applied to the CEC’17 benchmark functions test set, with the results proving the superior solution capabilities of the improved algorithm. With regard to the issue of PEMFC and PV cell parameter identification, both the accuracy and convergence speed of obtaining the optimal solution are significantly better in the improved HBA when compared to the original HBA. Additionally, this study compared the computational results of the improved HBA against optimized parameter data from other pieces of relevant literature. The results of this comparison indicated that the optimal solution obtained by the improved is also better than that of many other algorithms. In all, it is clear that the proposed modifications to the HBA can improve its computational efficiency, which will be a significant contribution to the power scheduling and prediction efficiency of energy management systems and green energy systems.

## Figures and Tables

**Table 1:** Algorithmic settings for computation.

Independent Run#	Iteration#	Agent#
30	1000	30

**Table 2:** Upper and lower bounds of parameter identification for PEMFC [40].

Fuel Cell Stack (NedStackPS6)							
Parameter	$\zeta_{1}$	$\zeta_{2}\times 10^{-3}$	$\zeta_{3}\times 10^{-5}$	$\zeta_{4}\times 10^{-4}$	λ	$R_{c}\times 10^{-4}$	b
Upper Bound	−0.8532	5	9.8	−0.954	24	8	0.5
Lower Bound	−1.19969	1	3.6	−2.60	10	1	0.0136

**Table 3:** Upper and lower bounds of parameter identification for photovoltaics [41].

RTC France Commercial Silicon PV Cell							
Parameter	*I_ph_* (A)	*I*_*sd*1_ (μA)	*I*_*sd*2_ (μA)	*R_ser_* (Ω)	*R_sh_* (Ω)	$n_{1}$	$n_{2}$
Upper Bound	1	1	1	0.5	100	2	2
Lower Bound	0	0	0	0	0	1	1

**Table 4:** Search results of the CEC’17 function set using HBA and IHBA.

Function	Fitness	Basic HBA	Improved HBA
F1	Best	209.6551	100.6777
	Mean	3768.2806	4631.458
	Stsd.	3027.036	4122.978
F3	Best	300	300
	Mean	300	300
	Std.	5.55 × 10^−7^	1.41 × 10^−12^
F4	Best	400.1116	400.0011
	Mean	404.5097	401.095
	Std.	10.0786	0.67054
F5	Best	505.9698	506.9648
	Mean	517.5666	525.2659
	Std.	6.5071	12.7717
F6	Best	600.0006	600.0252
	Mean	600.265	602.1771
	Std.	0.67757	2.1659
F7	Best	719.2101	717.709
	Mean	737.239	741.2192
	Std.	10.2652	12.7924
F8	Best	807.9597	809.9496
	Mean	818.473	818.1881
	Std.	6.0285	6.6923
F9	Best	900	900.0895
	Mean	904.2165	914.004
	Std.	8.7576	18.6761
F10	Best	1441.924	1260.2131
	Mean	2005.8985	1964.1629
	Std.	467.7276	504.9231
F11	Best	1104.9748	1101.9932
	Mean	1117.8216	1163.8663
	Std.	18.3487	110.3798
F12	Best	2650.4972	2108.2747
	Mean	16,696.9159	21,519.8767
	Std.	15,489.0404	16,919.6423
F13	Best	1648.4958	1313.6664
	Mean	7877.9512	6528.1912
	Std.	7433.3104	4919.5201
F14	Best	1438.4591	1433.2795
	Mean	1509.2567	1503.7298
	Std.	53.4582	39.6003
F15	Best	1503.7858	1526.7019
	Mean	1660.4571	1769.9305
	Std.	104.1281	212.0772
F16	Best	1601.3582	1600.9905
	Mean	1723.2108	1719.7966
	Std.	158.1934	92.3512
F17	Best	1714.02	1718.9231
	Mean	1744.3651	1748.2927
	Std.	29.8761	25.8519
F18	Best	2001.399	2049.0235
	Mean	9254.6979	8293.0935
	Std.	8758.0398	9346.8135
F19	Best	1906.0762	1916.936
	Mean	2016.1874	3621.1217
	Std.	100.837	5661.1408
F20	Best	2006.8411	2016.1532
	Mean	2071.7073	2064.2511
	Std.	73.3149	56.0847
F21	Best	2200	2200
	Mean	2301.1063	2291.4835
	Std.	55.098	59.1305
F22	Best	2300.3982	2237.6807
	Mean	2302.3966	2301.379
	Std.	1.6882	12.1854
F23	Best	2612.8207	2608.0425
	Mean	2631.4024	2632.7816
	Std.	18.0681	14.1716
F24	Best	2739.5949	2500
	Mean	2759.0692	2737.1695
	Std.	12.8938	81.2033
F25	Best	2897.9379	2600.1524
	Mean	2929.2155	2925.7454
	Std.	23.486	63.6884
F26	Best	2800	2600
	Mean	3030.5947	3028.2535
	Std.	329.9593	306.0842
F27	Best	3090.3325	3093.5835
	Mean	3120.7443	3133.8102
	Std.	48.958	40.3056
F28	Best	2800	3100
	Mean	3400.6286	3375.285
	Std.	261.5139	194.0603
F29	Best	3160.0644	3137.516
	Mean	3252.4807	3225.6448
	Std.	65.7085	84.2785
F30	Best	4885.6424	3561.792
	Mean	2,682,074.603	1,650,759.864
	Std.	5,471,052.797	3,461,462.501
Score	Best	13	18
	Mean	13	17
	Std.	17	12

**Table 5:** Results of PEMFC parameter identification with IHBA and eight other algorithms.

ALGO	ξ_1_	ξ_2_	ξ_3_	ξ_4_	λ	$\Omega_{c}$	b	SSE
IHBA	−0.85546	2.636 × 10^−3^	5.25 × 10^−5^	−9.54 × 10^−5^	12.5743308	10^−4^	1.36 × 10^−2^	2.06555
HBA	−0.8532	2.42242 × 10^−3^	3.7701 × 10^−5^	−9.54 × 10^−5^	12.5743308	10^−4^	1.36 × 10^−2^	2.06555691
IABC [42]	−0.989151	3.55443 × 10^−3^	8.39696 × 10^−5^	−9.54002 × 10^−5^	11.8775	10^−4^	1.36025 × 10^−2^	2.9848
PSO [42]	−0.927807	3.59632 × 10^−3^	9.8 × 10^−5^	−9.54 × 10^−5^	24	6.76895 × 10^−4^	1.36 × 10^−2^	5.56449
BO [42]	−0.9704	3.70109 × 10^−3^	9.8 × 10^−5^	−9.54679 × 10^−5^	11.8781	10^−4^	1.36 × 10^−2^	2.9849
MLNNA [43]	−1.0977288	3.1439 × 10^−3^	3.83 × 10^−5^	−9.54 × 10^−5^	13.0947079	0.1	1.36 × 10^−2^	2.0791657
WOA [43]	−0.8532	3.2673 × 10^−3^	9.8 × 10^−5^	−9.54 × 10^−5^	13.2263552	0.1002529	1.72465 × 10^−2^	2.1043370
BES [44]	−1.149035	3.3487 × 10^−3^	3.60 × 10^−5^	−9.54 × 10^−5^	13.09754	10^−4^	1.36 × 10^−2^	2.07974
SSO [45]	−1.017	2.315 × 10^−3^	5.24 × 10^−5^	−1.2815 × 10^−5^	18.855	7.5 × 10^−4^	1.36 × 10^−2^	7.1889

**Table 6:** Experimental values and terminal voltage calculations for PEMFC with HBA and IHBA.

Experimental Data		Basic HBA	Improved HBA
$I_{t}$ (A)	$V_{t}$ (V)	Computed $V_{t}$ (V)	
2.25	61.64	62.3558	62.3558
6.75	59.57	59.7818	59.7818
9	58.94	59.0504	59.0504
15.75	57.54	57.4982	57.4982
20.25	56.8	56.7195	56.7195
24.75	56.13	56.0462	56.0462
31.5	55.23	55.1589	55.1589
36	54.66	54.6222	54.6222
45	53.61	53.6345	53.6345
51.75	52.86	52.9453	52.9453
67.5	51.91	51.4403	51.4403
72	51.22	51.0277	51.0277
90	49.66	49.4184	49.4184
99	49	48.6270	48.6270
105.8	48.15	48.0308	48.0308
110.3	47.52	47.6361	47.6361
117	47.1	47.0473	47.0473
126	46.48	46.2521	46.2521
135	45.66	45.4494	45.4494
141.8	44.85	44.8364	44.8364
150.8	44.24	44.0146	44.0146
162	42.45	42.9721	42.9721
171	41.66	42.1157	42.1157
182.3	40.68	41.0137	41.0137
189	40.09	40.3446	40.3446
195.8	39.51	39.6526	39.6526
204.8	38.73	38.7149	38.7149
211.5	38.15	37.9996	37.9996
220.5	37.38	37.0139	37.0139

**Table 7:** Photovoltaic parameters identified by IHBA and eight different algorithms.

ALGO	$I_{ph}$ (A)	$I_{sd1}$ (μA)	$I_{sd2}$ (μA)	$R_{ser}$ (π)	$R_{sh}$ (π)	$n_{1}$	$n_{2}$	RMSE
IHBA	0.760795551	1	0.093593878	0.037908261	55.85481567	1.840391678	1.38405544	8.545 × 10^−4^
HBA	0.760856985	0.173843326	1	0.037459716	53.82679743	1.429106962	2	8.651 × 10^−4^
ChOA [46]	0.7607739	0.2229	0.727181	0.036377	55.426432	1.451227	2	9.7201 × 10^−4^
WHHO [46]	0.7607745	0.2289	0.727181	0.036335	55.426432	1.451338	2	9.7202 × 10^−4^
EHHO [47]	0.760769017	0.586184	0.240965	0.036598831	55.63943956	1.968451449	1.456910409	9.83606 × 10^−4^
ABC [47]	0.7608	0.0407	0.2874	0.0364	53.7804	1.4495	1.4885	9.861 × 10^−4^
GAMS [48]	0.760781	0.225974	0.749479	0.036740	55.485441	1.451021	2.000000	9.824848 × 10^−4^
AHA [49]	0.760780604268300	0.232255097648817	0.696348169771192	0.0367112661935343	55.3572707250706	1.45330976194524	1.99999999173321	9.82505533909522 × 10^−4^
EJAYA [50]	0.76078	0.22597	0.74934	0.03674	55.48509	1.45102	2.00000	9.8248 × 10^−4^

**Table 8:** Terminal current values calculated by HBA and IHBA.

Experimental Data		Basic HBA	Improved HBA
$V_{t}$ (V)	$I_{t}$ (A)	Computed $I_{t}$ (A)	
−0.2057	0.764	0.7641	0.7640
−0.1291	0.762	0.7627	0.7626
−0.0588	0.7605	0.7614	0.7613
0.0057	0.7605	0.7602	0.7602
0.0646	0.76	0.7591	0.7591
0.1185	0.759	0.7581	0.7581
0.1678	0.757	0.7571	0.7572
0.2132	0.757	0.7562	0.7562
0.2545	0.7555	0.7551	0.7552
0.2924	0.754	0.7536	0.7537
0.3269	0.7505	0.7513	0.7513
0.3585	0.7465	0.7472	0.7472
0.3873	0.7385	0.7399	0.7399
0.4137	0.728	0.7273	0.7272
0.4373	0.7065	0.7070	0.7069
0.459	0.6755	0.6754	0.6753
0.4784	0.632	0.6310	0.6309
0.496	0.573	0.5722	0.5721
0.5119	0.499	0.4998	0.4997
0.5265	0.413	0.4137	0.4136
0.5398	0.3165	0.3174	0.3172
0.5521	0.212	0.2119	0.2117
0.5633	0.1035	0.1021	0.1020
0.5736	−0.01	−0.0084	−0.0084
0.5833	−0.123	−0.1245	−0.1243
0.59	−0.21	−0.2066	−0.2061
−0.2057	0.764	0.7641	0.7640
−0.1291	0.762	0.7627	0.7626
−0.0588	0.7605	0.7614	0.7613

## Data Availability

The data that support the findings of this study are available on request from the corresponding author.
